# Differential ATAC-seq and ChIP-seq peak detection using ROTS

**DOI:** 10.1093/nargab/lqab059

**Published:** 2021-07-02

**Authors:** Thomas Faux, Kalle T Rytkönen, Mehrad Mahmoudian, Niklas Paulin, Sini Junttila, Asta Laiho, Laura L Elo

**Affiliations:** Turku Bioscience Centre, University of Turku and Åbo Akademi University, Tykistökatu 6, 20520, Turku, Finland; Turku Bioscience Centre, University of Turku and Åbo Akademi University, Tykistökatu 6, 20520, Turku, Finland; Institute of Biomedicine, University of Turku, Kiinamyllynkatu 10, 20014, Finland; Turku Bioscience Centre, University of Turku and Åbo Akademi University, Tykistökatu 6, 20520, Turku, Finland; Department of Future Technologies, University of Turku, FI-20014 Turku, Finland; Turku Bioscience Centre, University of Turku and Åbo Akademi University, Tykistökatu 6, 20520, Turku, Finland; Turku Bioscience Centre, University of Turku and Åbo Akademi University, Tykistökatu 6, 20520, Turku, Finland; Turku Bioscience Centre, University of Turku and Åbo Akademi University, Tykistökatu 6, 20520, Turku, Finland; Turku Bioscience Centre, University of Turku and Åbo Akademi University, Tykistökatu 6, 20520, Turku, Finland; Institute of Biomedicine, University of Turku, Kiinamyllynkatu 10, 20014, Finland

## Abstract

Changes in cellular chromatin states fine-tune transcriptional output and ultimately lead to phenotypic changes. Here we propose a novel application of our reproducibility-optimized test statistics (ROTS) to detect differential chromatin states (ATAC-seq) or differential chromatin modification states (ChIP-seq) between conditions. We compare the performance of ROTS to existing and widely used methods for ATAC-seq and ChIP-seq data using both synthetic and real datasets. Our results show that ROTS outperformed other commonly used methods when analyzing ATAC-seq data. ROTS also displayed the most accurate detection of small differences when modeling with synthetic data. We observed that two-step methods that require the use of a separate peak caller often more accurately called enrichment borders, whereas one-step methods without a separate peak calling step were more versatile in calling sub-peaks. The top ranked differential regions detected by the methods had marked correlation with transcriptional differences of the closest genes. Overall, our study provides evidence that ROTS is a useful addition to the available differential peak detection methods to study chromatin and performs especially well when applied to study differential chromatin states in ATAC-seq data.

## INTRODUCTION

Chromatin states can be seen as the collection of proteins or histone modifications that regulate the openness and activity of a given chromatin region (1,2). Dynamic regulation of chromatin states drives changes in gene transcription and consequently in cellular phenotypes (3), and proceeds largely through modification of chromatin-associated histone proteins to open or close chromatin access (4–6). These histone modifications can be studied with chromatin immunoprecipitation followed by sequencing (ChIP-seq) (6) that has provided substantial insights on gene regulation. Other methods have been developed to reveal the open or closed chromatin states, such as DNase I hypersensitive sites sequencing (DNase-seq) (7), formaldehyde-assisted isolation of regulatory elements (FAIRE-seq) (8) and assay for transposase accessible chromatin followed by high-throughput sequencing (ATAC-seq) (9). ATAC-seq has gained popularity due to the fact that it requires considerably less genetic material than previous methods (10).

The output of both ChIP-seq and ATAC-seq analysis consists of reads enriched in genomic locations (also called peaks) representing the presence of a protein in the case of ChIP-seq or open chromatin state in the case of ATAC-seq. Thus, methods first developed for ChIP-seq data analysis, such as MACS2 (11) and HOMER (12), have also been used to identify open chromatin regions from ATAC-seq data (13,14).

In addition to peak calling, it is of specific importance to find significant differences in chromatin states between the biological conditions or groups of interest from the ChIP-seq and ATAC-seq data. A major challenge in such differential ChIP-seq and ATAC-seq data analysis is the large search space, as it is not limited only to, e.g. protein coding genes as in gene expression analysis, but in theory, open chromatin and protein binding events take place across most of the genome. Additionally, the range of the signal does not have intrinsic boundaries like the 0 to 100% in DNA methylation, as there is no theoretical upper limit for the read enrichments. An additional challenge in detecting the differential states in the context of ChIP-seq also lies in the amount of noise generated by the immunoprecipitation step, which makes it difficult to detect subtle changes between conditions (15,16). Several methods have been developed to solve such challenges and to detect differential enrichment in reads (also called differential peaks) (17–19).

The existing methods for differential peak calling can be classified into one-step methods that inherently include the initial peak calling, and two-step methods that require the prior use of a separate peak caller to produce the peaks for differential analysis. The one-step methods can be further separated into sliding window methods (e.g. DiffReps (19) or PePr (18)), and segmentation methods such as Hidden Markov Models (HMM) [e.g. THOR (17)]. The sliding window approaches use a user-defined window to scan the genome for enrichment. These approaches can be sensitive to the window size selected; a too wide window might miss local changes and a too narrow window might miss global scale changes (15,17). While HMM-based methods enable flexibility on the size of detected regions, they can be sensitive to small-scale changes in the signal (15). The two-step methods [e.g. DiffBind (20)] require the candidate peaks to be defined by an external peak calling software, such as MACS2 (11), HOMER (12) or SICER (21). Concordantly, they are restricted to the search-space defined by the candidate peaks with the chosen peak caller. While several methods have been developed to tackle the differential peak calling problem, the challenge remains that the overlap between the results from different methods is often small and it is difficult to evaluate the true positives (15,16).

ATAC-seq is intensively applied in the chromatin studies because of its ease of use (no antibody steps and small sample quantities), but the differential peak calling methods have been previously compared only using ChIP-seq data (15–20), constituting a specific need for a comparison that also applies these methods to ATAC-seq data. The ease of use combined with decreasing sequencing cost has allowed inclusion of growing numbers of experimental replicates especially in ATAC-seq studies. While the early ChIP-seq studies often included only two or even just one replicate, nowadays the need for at least three replicates in ChIP-seq (and ATAC-seq) studies is widely recognized (22). In addition to enabling better separation of consistent biological occurrences from random events, a higher number of biological replicates helps to mitigate the effect of background noise which is often high in ChIP-seq studies due to the non-specific binding. While some of the early differential peak callers allowed only two replicates, e.g. ODIN (23) and MAnorm (24), later iterations are not limited to a certain number of replicates.

Here, we introduce the application of reproducibility optimized test statistic (ROTS) (25) for robust differential peak calling on chromatin data with multiple replicates. A major advantage of ROTS is its efficient use of replicates to optimize the reproducibility of the results by bootstrapping the data. Previously ROTS has shown good performance in the context of differential gene expression (26), differential DNA methylation sequencing (27) and mass spectrometry proteomics (28). Here we apply ROTS to differential peak calling in ChIP-seq and ATAC-seq data and systematically compare its performance to five commonly used methods DiffBind (20), DiffReps (19), MAnorm2 (29), PePr (18) and THOR (17) using both ChIP-seq and ATAC-seq datasets. We rigorously investigate the intensity and breadth of the called differential peaks and also estimate the performance of the methods by correlating the fold-changes of the differential chromatin states with the differential gene expression fold-changes of the nearest genes. Importantly, our study is the first to compare the differential peak calling methods simultaneously with both ChIP-seq and ATAC-seq data.

## MATERIALS AND METHODS

### General description of ROTS

The ROTS is an approach that is based on investigating the inherent characteristics of the data and thus is able to free itself from any distributional assumptions (25). Specifically, ROTS maximizes the reproducibility *z*-statistic ${Z_k}( {{d_\alpha }} )$over parameters $\alpha$ and the top list size *k* by considering the reproducibility of the *k* top-ranked features ${R_k}( {{d_\alpha }} )$ using a family of *t*-type statistics ${d_\alpha }$, in pairs of bootstrapped dataset:

$$\begin{equation*}{Z_k} \left( {{d_\alpha }} \right) = \frac{{{R_k}\left( {{d_\alpha }} \right) - R_k^0\left( {{d_\alpha }} \right)}}{{{s_k}\left( {{d_\alpha }} \right)}} \end{equation*}$$

${R_k}( {{d_\alpha }} )$ and $R_k^0$(${d_\alpha }$) are respectively the reproducibility of the bootstrapped and randomized data and ${s_k}( {{d_\alpha }} )$ is the estimated standard deviation of the bootstrap distribution. ${R_k}( {{d_\alpha }} )$represents the average overlap of the *k* top-ranked features over *B* pairs of bootstrap datasets. The reproducibility for each pair *b* of bootstrap data matrices ($D_1^{( b )}$, $D_2^{( b )}$) is calculated as:

$$\begin{equation*}R_k^{\left( b \right)} \left( {{d_\alpha }} \right) = \frac{{\# \{ g|{r_g}\left( {\alpha ,D_1^{\left( b \right)}} \right) \le k, {r_g}\left( {\alpha , D_2^{\left( b \right)}} \right) \le k\} }}{k}\end{equation*}$$

where ${r_g}( {\alpha ,D_i^{( b )}} )$ denotes the rank of feature *g* in data $D_i^{( b )}$ with the statistic ${d_\alpha }$ and *#S* is the cardinality of set *S*.

The test statistics ${d_\alpha }$ for a genomic feature of interest $g$ (here chromatin region) is defined as:

$$\begin{equation*}{d_\alpha } \left( g \right) = {\rm{ }}\frac{{\left| {\bar{x}_{g{\rm{ }}}^i - \bar{x}_{g{\rm{ }}}^j} \right|}}{{{\alpha _1} + {\alpha _2}{s_g}}},\end{equation*}$$

where $\bar{x}_{g\ }^i$ and $\bar{x}_{g\ }^j$ are the average number of reads of feature $g$ in the experimental conditions $i$ and $j$ and ${s_g}$ represents the estimated standard error.

The input required by ROTS is a matrix of preprocessed and normalized read counts with columns constituting the samples and rows constituting the enriched peak regions determined using a peak caller. ROTS R package and a thorough manual are available through Bioconductor at https://bioconductor.org/packages/ROTS.

### Differential peak calling workflow for ROTS

Before the differential peak calling with ROTS, we first performed the initial peak calling for each sample using MACS2 (11), which is widely used and has shown good performance in independent comparisons (30). As recommended in (31), we performed the peak calling for each condition on the pooled reads of the available samples. Next, a candidate peak list was compiled based on the sample-level peak calls by taking the union of the peaks present across the sample set and merging the overlapping peaks together. After this, a count matrix was produced that contained the read count of each candidate peak for each sample. To normalize the reads, we used the median of ratios available in the DESeq2 R/Bioconductor package (32), as it is robust in the case of outliers, but also in the case of imbalance in the number of peaks between conditions.

### Overview of the compared methods

We selected five differential peak calling methods to be compared to our proposed ROTS-based approach (Table 1): DiffBind (20), MAnorm2 (29), diffReps (19), PePr (18) and THOR (17). The methods were selected according to their popularity, their support for the use of replicates, and their ability to detect differences in signal associated to chromatin state which are known to be characteristically broad genomic regions. When comparing the methods, we put ourselves in the place of a typical user that uses the methods ‘out of the box’, with the parameters recommended by the developers. DiffBind is a two-step method that uses an external peak caller (in our study MACS2) and the R/Bioconductor package DEseq2 (33) to normalize the data with the median of ratios method (32) and to perform the statistical testing. MAnorm2 is a two-step method that uses an external peak caller (in our study MACS2), normalises the data based on a linear fit of M- and A-values (respectively log2 fold change and mean log2 read count), and performs statistical testing by adopting the modeling strategy of limma (34). DiffReps is a one-step method that uses a sliding window to scan the genome for differences, a linear method for normalization, and an exact negative binomial test for determining the differences. PePr is a one-step method that uses a sliding window to find genomic regions with differences, TMM for normalization, and negative binomial distribution for read modeling. THOR is a one-step method that normalizes the data using the Trimmed Mean of *M*-values (TMM) (35) and uses an HMM with three hidden states to find regions with differences between conditions.

**Table 1.** Overview of the methods compared in the study

Tool	Language	Input	Peak calling	Default normalization	Statistical test	Reference
one-step						
THOR	Python	Reads (*.bam)	Not required	TMM	HMM with a three state topology	Allhoff, M. et al. (2014)
diffReps	Perl	Reads (*.bam)	Sliding window approach	Linear normalization	Exact negative binomial test	Shen, L. *et al.* (2013)
PePr	Python	Reads (*.bam)	Sliding window approach	TMM	Binomial distribution	Zhang, Y. et al. (2014)
two-step						
DiffBind	R	Reads (*.bam) Peaks (*.bed)	Peak caller required (e.g. MACS2)	DEseq2	DEseq2 (default) DEseq edgeR	R. Stark, G. B. (2011)
ROTS	R	Reads (*.bam) Peaks (*.bed)	Peak caller required (e.g. MACS2)	DEseq2	differential analysis performed with ROTS	Suomi, T *et al.* (2017)
MAnorm2	R	Reads (*.bam) Peaks (*.bed)	Peak caller required (e.g. MACS2)	Remove MA trend from common peaks	Differential analysis adapted from limma	Tu, S *et al.* (2020)

### Overview of the datasets

We selected five datasets for our study: two biological datasets were based on ATAC-seq [Interferon response (IFN) and Yellow Fever vaccine (YF)], two were from ChIP-seq studies (Rheumatoid Arthritis H3K4me3 and H3K36me3), while the fifth was a synthetic dataset. The four biological datasets (Table 2) were selected for their relatively high number of replicates (>5) and for the presence of matching RNA-seq data. The synthetic dataset was from an earlier differential peak detection tool comparison that modeled H3K36me3 binding (15). It was generated on the basis of top 20 000 detected peaks from a reference sample using MACS2, which were then divided into two groups for further simulation: 10 000 true differential peaks and 10 000 non-differential peaks. Using the reads within these peaks, a treatment sample was simulated based on the reference sample by downsampling the reads of the true differential peaks across 10 different intensity categories (from 100% intensity to 10% intensity, 1000 peaks per intensity category). For both the treatment and the reference samples, another layer of variation was finally added by simulating biological noise (15). For our comparison study, we further downsampled the reference and treatment samples five times by a random percentage between 10 and 30% to create five simulated biological replicates per condition. The statistical comparison was always done for the same peak region between the sample groups and as such was not affected by the size of the peak.

**Table 2.** Overview of the biological datasets used in the study

Epigenetic mark	Condition	Subject	Replicates per condition	Reference	GEO accession number
Open chromatin	Yellow fever (YF)	CD8 Tcells	8	Akondy *et al.*, 2017 (52)	GSE101609
Open chromatin	Interferon response (IFN)	CD14+ monocyte derived macrophages	6	Park *et al.*, 2017 (53)	GSE100383
H3K4me3	Rheumatoid arthritis (RA)	Fibroblast like synoviocytes	10	Ai *et al.*, 2018	GSE112655
H3K36me3	Rheumatoid arthritis (RA)	Fibroblast like synoviocytes	10	Ai *et al.*, 2018 (54)	GSE112655

### ChIP-seq and ATAC-seq pre-processing, peak calling and differential peak calling

All biological datasets were downloaded as sra files from the Sequence Read Archive (SRA) and converted to fastq format with fastq-dump tool from the SRA Toolkit (36). Reads were aligned to human hg19 reference genome (37) derived from UCSC Genome Browser using Bowtie2 (38) with default settings. Reads with mapping quality below 15 and the reads in the regions of low complexity or high repeatability on the genome, as listed by the ENCODE consortium hg19 (39), were removed with samtools 1.2 (40). For the two-step methods, we pooled the reads across the samples for each condition as recommended in (31). Peak calling was performed using MACS2 (11) with significance cut-off ‘-q 0.01’, and for ATAC-seq option -f BAMPE was defined. We chose to use the ‘narrow’ peak calling option for three datasets (YF ATAC-seq, IFN ATAC-seq and H3K4me3 ChIP-seq) and the ‘broad’ option for H3K36me3 ChIP-seq in accordance with the ENCODE guidelines (https://www.encodeproject.org/chip-seq/histone). For the comparative purpose of this study, the ChIP-seq datasets were compared without subtracting the input chromatin sample, because as such the detection of differential binding between two conditions does not require an input ChIP-seq control (31,41). The differential peak calling was run with each tool according to the settings recommended by the developers in either publication, vignette or tutorial. The samples from the synthetic data were all created using the same set of reads and normalization for sequencing depth was not required. Hence, for synthetic data we ran ROTS, MAnorm2, PePr and THOR without normalization, whereas diffReps, and DiffBind were run with their default normalization. We initially tested the DiffBind method with both DESeq2 and edgeR and observed that edgeR introduced a marked number of false positives. Based on this we continued running DiffBind with DESeq2.

### Evaluation of differential peak calling on synthetic data

We used the GenomicRanges R/Bioconductor package (42) to detect the overlap between the significant peaks (FDR < 0.05) called by the different methods and the true differential and non-differential peaks. An overlap of 1 bp and an overlap of at least 25% of the true peaks were tested and produced similar results.

### Differential gene expression in RNA-seq data

Each of the biological ATAC-seq/ChIP-seq datasets included in our study contained matching gene expression data. For ATAC-seq datasets, we used the normalized gene expression data available from the original studies; in the YF dataset the available read counts had been corrected for batch effect with ComBat (43), and in the IFN dataset the raw counts had been normalized by means of fragments per kilobase of exon per million fragments mapped (FPKM). For the rheumatoid arthritis (RA) datasets, only raw read counts were available, so we normalized them with TMM and converted them to counts per million. We used ROTS (44) to perform the differential expression analysis and calculated the differential expression fold-change for each dataset from the difference in means of read count values.

### Evaluation of differential peak calling on biological data

Evaluation of differential peak calling in real biological data is challenging, as there is no existing biological gold standard. To circumvent this, we used the correlation to gene expression data as suggested previously (23,45–48). This approach is based on the assumption that open chromatin and activating histone binding domains correlate with expression levels of the surrounding genes. By looking at the correlation of fold-changes in differential chromatin states and fold-changes in transcription of the closest gene, the performance of the differential peak calling can then be approximated. For this, we first ranked the differential peaks from each method according to their FDR. Peaks were then annotated to their nearest genes. If multiple peaks were annotated to one gene, the fold-change of differential peaks for the gene was calculated by taking the average fold-change of all the peaks annotated to the gene. The Pearson correlation between the log10 fold-change of differential binding and log10 fold-change of the differential gene expression was then calculated iteratively using an increasing number of top ranked differential peaks. Our decision to focus on 2000 top peaks was a compromise considering both the number of called peaks, placing emphasis on the most significantly detected peaks (Table 3), as well as on the number of differentially regulated genes (FDR < 0.05 and FC > 2) in the corresponding gene expression data that varied in the range of 325–3186. The median breadth of the peaks between one-step methods and two-step methods was compared with Wilcoxon test.

**Table 3.** The number of significant differential peaks (FDR < 0.05) detected by the methods

	Two-step			One-step		
	ROTS	DiffBind	MAnorm2	diffReps	PePr	THOR
YF ATAC-seq	2017	8736	3816	9168	1955	36 009
IFN ATAC-seq	37 630	40 001	32 362	57 209	44 143	91 118
RA H3K4me3	1913	3111	10	21 443	1072	17 343
RA H3k36me3	11	25	0	27 549	1077	17 483

## RESULTS

### Comparing differential peak calling methods in synthetic data

**Figure 1.**
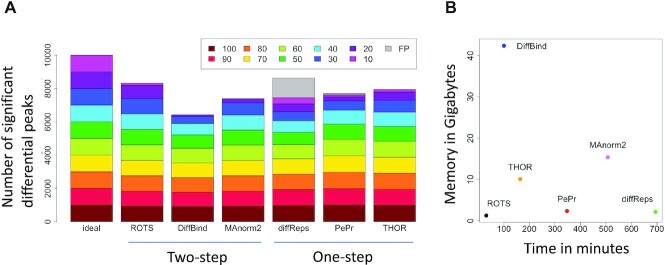
Performance on synthetic data. (**A**) The number of significant differential peaks detected by each method compared to the theoretical ideal. The colors represent the different intensity categories from 100% intensity to 10% intensity, reflecting the differences between the sample groups. The gray color denotes false positive peaks. Ideally, 1000 differential peaks are detected per intensity category. (**B**) The computing time and the memory consumption across the methods.

We compared the performance of ROTS with five popular differential peak detection methods; DiffBind, diffReps, MAnorm2, PePr and THOR, on a synthetic dataset adapted from a previous study (15), containing a mixture of true differential and non-differential peaks, 10 000 of each, across 10 different intensity categories. When comparing the overlap between the significant peaks (FDR < 0.05) called by the different methods and the true differential and non-differential peaks, all the methods detected differential peaks well when the simulated differences were considerable, i.e. between 60 and 100% difference in signal intensity between the conditions (Figure 1A). With smaller differences, THOR, PePr and especially ROTS were able to recover larger proportions of the true peaks compared to the other methods, which detected few or no peaks with differences in signal intensity below 30%. Overall, the methods reported very few of the non-differential peaks as significant, with the exception of diffReps which called a relatively large number of false positive peaks (1178 false positives). The sensitivity and specificity are also illustrated across the methods as receiver operating characteristic curves (Supplementary Figure S1), highlighting the good accuracy of especially ROTS, MAnorm2 and THOR.

We also tested the speed and the memory consumption of the methods when run on a modern computer cluster managed by the open-source Simple Linux Utility for Resource Management. In our inspection, ROTS was by far the fastest and the most memory efficient method (Figure 1B). DiffBind on the other hand showed particularly large memory consumption, followed by MAnorm2. MAnorm2 was the second slowest and diffReps the slowest of the compared methods, the latter being more than 20 times slower than ROTS.

### Number of differentially called peaks and overlap between methods in real ATAC-seq and ChIP-seq data

The number of significant peaks (FDR < 0.05) reported varied considerably between the different methods within each dataset (Table 3). THOR and diffReps overall reported many more differential peaks than the other methods across the datasets, typically in the order of tens of thousands. With PePr the number of detections varied greatly across the datasets; it reported a relatively high number of differential peaks with IFN ATAC-seq dataset (44143 peaks), while for the other datasets it called a relatively lower number of differential peaks (<2000 peaks). The two two-step methods, ROTS, DiffBind and MAnorm2 reported comparable numbers of differential peaks, with the exception that in H3K4me3 data MAnorm2 detected only 10 peaks with FDR < 0.05 (versus ROTS 1913 peaks and DiffBind 3111 peaks). The one-step methods diffReps and especially THOR reported a significantly larger number of differential peaks across the datasets compared to the two-step methods. Largest number of differential peaks were consistently detected in the IFN ATAC-seq by all methods, while especially the two-step methods showed only very few differential peaks in the RA H3K36me3 ChIP-seq dataset.

**Figure 2.**
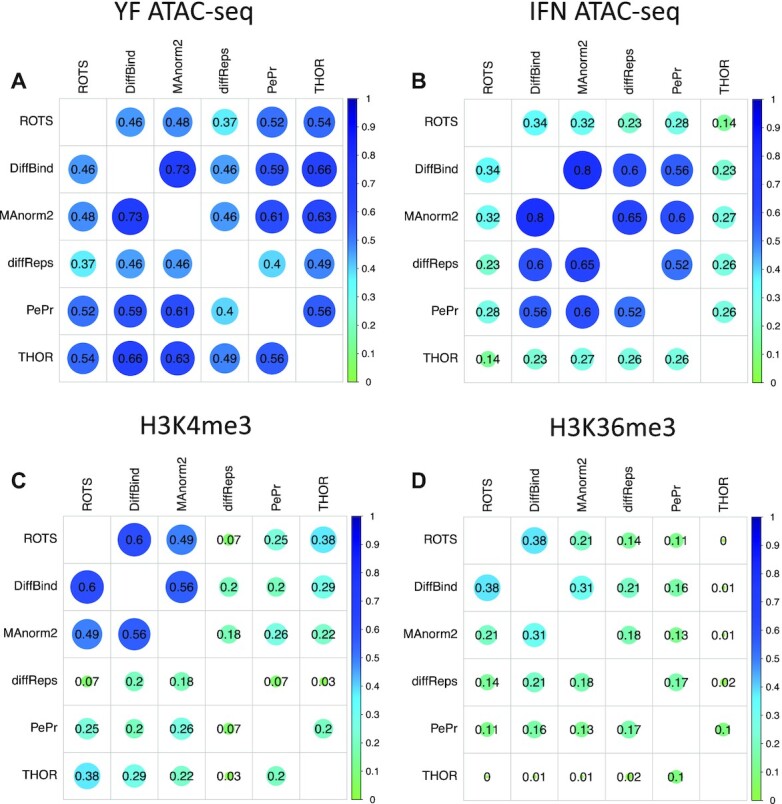
Overlap of the top 2000 detected significant differential peaks across the methods and biological datasets. Proportion of detected peaks overlapping between each pair of methods in (**A**) Yellow Fever ATAC-seq, (**B**) IFN ATAC-seq, (**C**) RA H3K4me3 ChIP-seq and (**D**) RA H3K36me3 ChIP-seq dataset.

Next, we compared the overlap of the most significant differential peaks between the methods across each of the biological datasets, with focus on top 2000 peaks (Figure 2). Overlap between the methods was generally higher in the two ATAC-seq datasets compared to the ChIP-seq datasets where poor overall overlap was observed especially in the H3K36me3 dataset. The two-step methods ROTS and especially DiffBind and MAnorm2 showed a significant overlap with each other across the four datasets (32–80% in ATAC-seq datasets and 21–60% in ChIP-seq datasets). Compared to two-step methods the one-step methods in general showed lower overlap across the datasets (26–56% in ATAC-seq datasets and <17% in ChIP-seq datasets). Overall, the two-step methods correlated best with other methods of the same type, while with one-step methods the correlation pattern varied more across the datasets. For complementarity, similar correlation plots were also made available based on all significant differential peaks (FDR < 0.05) (Supplementary Figure S2).

### Comparison of width and intensity of the differential peaks

**Figure 3.**
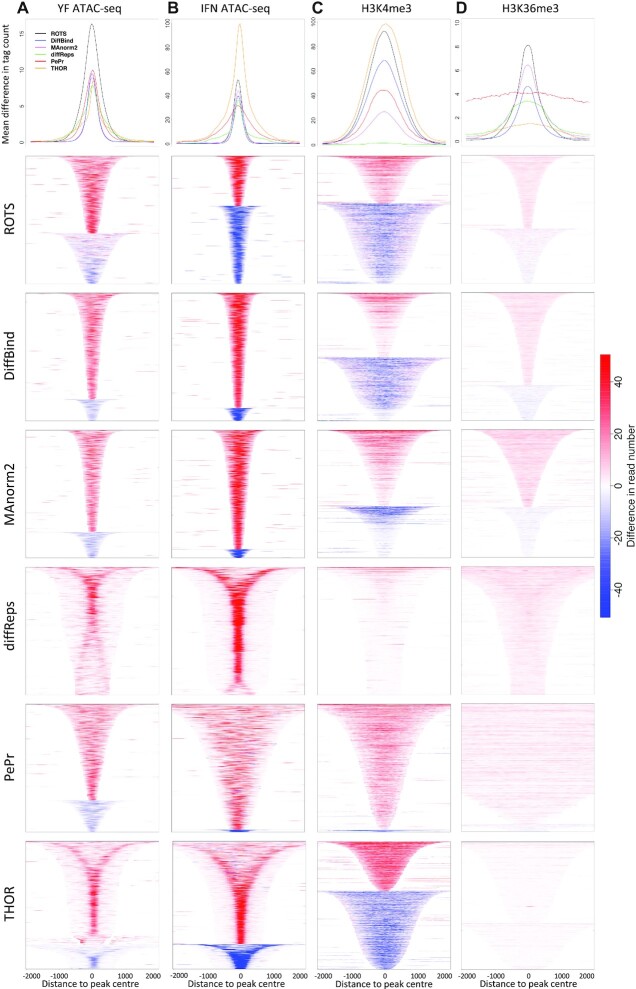
Width and enrichment intensity difference for the top 2000 differential peaks across the methods (rows) and datasets (columns). (**A**) Yellow fever ATAC-seq, (**B**) IFN ATAC-seq, (**C**) RA H3K4me3 ChIP-seq and (**D**) RA H3K36me3 ChIP-seq dataset. Panels on the first row display the absolute mean difference in read counts for the detected differential peaks with each method and dataset. The heatmaps display the difference in read counts and the direction of the change for each individual differential peak. Signal over the biological replicates per condition were averaged and the figures display a range of −2 kb and +2 kb from the center of the peak.

**Figure 4.**
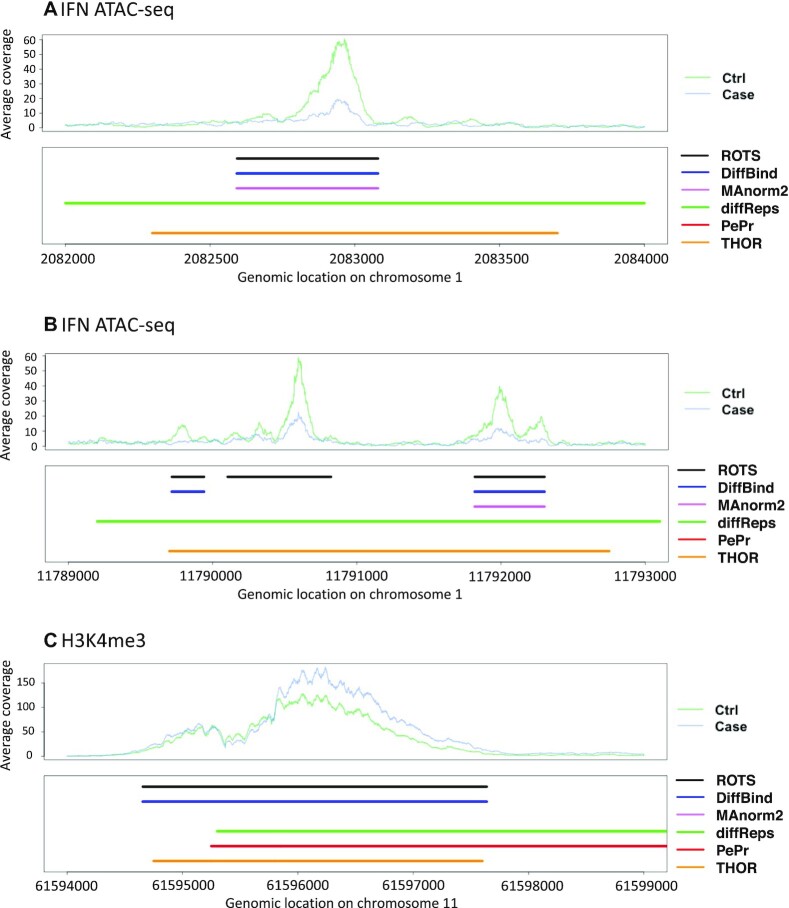
Selected representative examples of detected differential peaks in the genomic context. (**A**) and (**B**) IFN ATAC-seq, and (**C**) RA H3K4me3 dataset. The top panels display the average read count over each biological condition and the bottom panels mark the detected differential peak regions with each method.

In order to compare the shape of the peaks detected by the different methods, we assessed the intensity and width of the most significant differential peaks called by each method. We used heatmaps, average read counts across the peak regions and average peak widths to evaluate the 2000 most significant differential peaks (Figure 3 and Supplementary Figure S3). Additionally, we provide detailed visual examples of the detected differential peaks in the genomic context (Figure 4 and Supplementary Figure S4).

In the ATAC-seq datasets, all methods detected peaks with clear differences in read count values between the conditions (Figure 3A and B), while the detected differences were subtler in the histone modification ChIP-seq datasets (Figure 3C and D), especially in the RA H3K36me3 dataset. The heatmaps across the datasets generally depict that the top peaks of ROTS were most evenly distributed to increased or decreased intensity changes whereas the top peaks of diffReps and PePr included mostly unidirectional changes (Figure 3).

In the ATAC-seq datasets, the two-step methods (ROTS, DiffBind and MAnorm2) overall detected narrower peaks than the one-step methods (THOR, PePr and diffReps) (*P* < 2.2×10^–16^ for the top 2000 peaks) (Supplementary Figure S3). In the H3K36me3 dataset specifically PePr detected exceptionally broad peaks. With THOR and diffReps many of the detected peak regions in the ATAC-seq datasets consisted of a segment with higher intensity in the middle surrounded by much lower intensity segments on both sides, and some regions showed a bimodal peak pattern where two summits were observed on the two sides of the peak center (Figure 3A and B). In the RA H3K36me3 ChIP-seq dataset, ROTS, DiffBind and Manorm2 detected more tightly defined peaks than PePr and THOR which detected very broad peak domains with constant intensity and no clear borders (Figure 3D).

Visual inspection of the example loci further illustrates that results of diffReps, PePr and THOR, based on inbuilt candidate peak identification, included many lower intensity segments on both sides of the peak summit (Figure 4 and Supplementary Figure S4). THOR and diffReps especially reported many differential peaks that were typically detected as two separate peaks with ROTS and DiffBind (Figure 4B). In contrast, there were also examples of differential peaks with contiguous intensity summits reported as one differential peak by the two-step methods but several sub-peaks by THOR. Curiously, we also found specific cases where THOR did not report difference at the intensity summit of the actual peak but instead called two separate differential peaks on the two sides of the peak summit (Supplementary Figure S4B). In general, the sliding-window-based one-step methods (diffReps and PePr) tended to call regions broader than the visually apparent enrichment in reads (Figure 4 and Supplementary Figure S4), whereas THOR showed examples of both apparently over-extended calls (Figure 4A) and apparently accurate calls (Figure 4C). As expected, the two-step methods ROTS, DiffBind and MAnorm2 focused on identical peak regions identified based on the common set of initial candidate peaks from the MACS2 peak caller, with the exception that in H3K4me3 data MAnorm2 detected much less significant differential peaks compared to ROTS and DiffBind.

### Evaluation of the differential peaks using their correlation to gene transcription

In order to have experiment-specific external evaluation criteria, we used the closest phenotypic data, the transcriptomic data, available for each of the studied chromatin datasets as conceived previously (23). The underlining assumption with this approach is that both open chromatin detected by ATAC-seq and activating histone modification markers detected by ChIP-seq positively correlate with transcription of the nearest gene (45–48). Thus, we calculated the correlation between the fold-changes in chromatin states of the top significant peaks and the matching fold-changes in transcription of the closest gene and used this correlation as an approximation of the differential peak calling performance.

**Figure 5.**
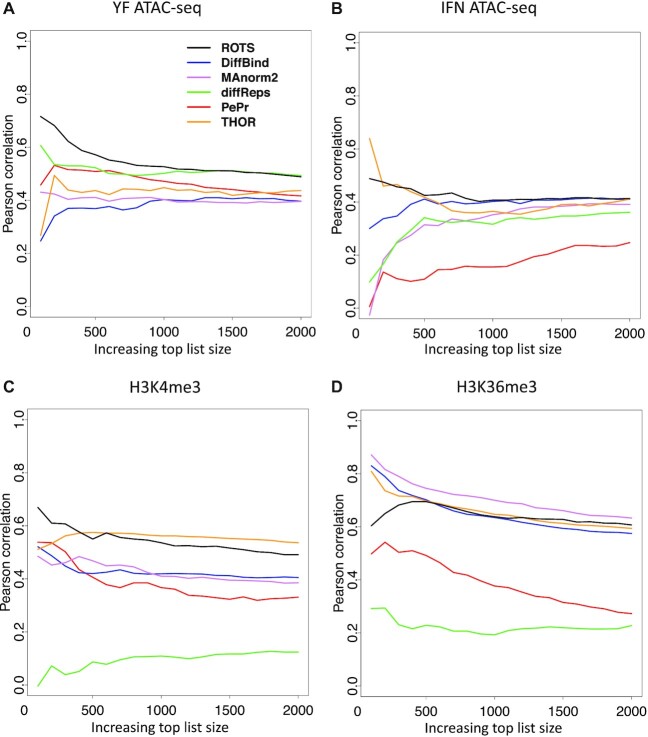
Pearson correlation between differential peak fold-change and gene expression fold-change for each method across an increasing number of top significant differential peaks in (**A**) Yellow Fever ATAC-seq, (**B**) IFN ATAC-seq, (**C**) RA H3K4me3 and (**D**) RA H3K36me3 dataset. The plot represents the correlation values with cut-offs starting from 100 peaks with increments of 100 peaks.

Differential peaks detected by ROTS consistently had a high overall correlation with differential transcription across the four datasets while the behavior of the other methods was more variable between the datasets (Figure 5A–D and Supplementary Figure S5A–D). Among the top 500 significant peaks, ROTS markedly showed the highest correlation in YF ATAC-seq and H3K4me3 datasets (Figure 5A and C). The sliding-window-based one-step methods PePr and diffReps showed the lowest overall correlation in other datasets, except in YF ATAC-seq (Figure 5A) where they had a high correlation close to that of ROTS. The two-step methods DiffBind and MAnorm2 had a similar performance with moderate correlations across the datasets, exceeding ROTS in only the H3K36me3 dataset (Figure 5D). THOR performed well with ChIP-seq data (Figures 5C and D), especially with H3K4me3.

## DISCUSSION

Accurate differential peak calling remains a challenge in the study of chromatin and histone modification states despite recent methodological advancements. ROTS is a computational tool that has been shown to work well in the statistical analysis of several types of high-throughput omics data by bootstrapping over the data to improve the reproducibility of the results and adapting the statistical test according to the intrinsic properties of the data (44). In this study, we applied ROTS for differential peak calling in two types of chromatin data, ChIP-seq and ATAC-seq, and compared it with five other widely used methods DiffBind (20), MAnorm2 (29), DiffReps (19), PePr (18) and THOR (17). Our results show that ROTS performed well both with synthetic data and biological data [two ATAC-seq datasets, two histone modification ChIP-seq datasets (H3K36me3 and H3K4me3)]. In the tested synthetic data, ROTS detected a larger portion of true differential peaks than the other methods, especially when the differences between the sample groups were relatively small (Figure 1). With the biological datasets, the two-step methods ROTS, DiffBind and MAnorm2 detected the enrichment boundaries more accurately than the one-step methods (Figures 3 and 4), while the differential chromatin states detected by ROTS showed higher correlations than DiffBind and MAnorm2 with the corresponding transcriptomic changes of the nearest genes (Figure 5), indicating detection of potentially biologically relevant differential chromatin states.

The differential peak detection software compared in this study can be defined as one-step methods that include the peak calling step as part of the method, and two-step methods that use a separate peak caller to produce the initial peaks for the differential analysis. By visual inspection of the mean signals (Figure 3) and examples of individual peaks (Figure 4) we observed that the two-step methods ROTS, DiffBind and MAnorm2 (with MACS2 called peaks) more accurately detected the apparent most differential enrichment than the one-step methods, which often detected broader peaks than the actual apparent read enrichments. The three one-step methods (diffReps, PePr and THOR) showed globally lower agreement between each other regarding the top significant peaks which is likely partially due to the fundamentally different underlying peak calling models, including HMM in THOR (17) or sliding window-based approaches in diffReps and PePr (18,19). We observed that the sliding window-based one-step methods (diffReps and PePr) detected broader regions than the visually inspected enrichment, which is concordant with previous notations (15,16). However, also the HMM-based THOR, allowing more flexibility in the selected window size, tended to call broader differential peak regions than visually evaluated enrichment boundaries in ATAC-seq datasets (Figure 3). This suggests that also one-step methods using HMMs can be sensitive to noise.

Based on our results, the two-steps methods relying on MACS2 input peaks were more consistent in finding the peaks with high-intensity enrichment in the middle of the peak than the one-step methods considered in this study. For instance, many of the THOR and diffReps (one-step methods) ATAC-seq peak regions showed a pattern where the highest signal of the broadest peaks was not in the middle of the peak but on the two sides of the peak centre (Figure 3), suggesting that a considerable proportion of the peaks detected by these methods are a composite of two summits. On the other hand, we also found examples of differential peaks with contiguous intensity summits reported as one differential peak by the two-step methods but as two separate peaks by the one-step methods. Interestingly, THOR also recovered cases of more complex patterns of differential sub-peaks inside a differential peak region (Figure 4C), suggesting that THOR could be useful in separating subpeaks reflecting, for example, sense versus anti-sense transcripts or alternative transcripts (49–51). Overall our findings extend the previous notion that MACS2 peak caller accurately detects peaks (30) in the context of differential peak calling by showing that it tends to detect peak borders more accurately compared to the peak calling steps by the one-step methods.

Finally, we extended our comparison to correlations between the fold-changes in chromatin state signals and fold-changes in transcription of the closest gene in each matched dataset. We used this correlation as an approximation of differential peak calling performance as previously conceived (23). The limitation of this approach is that it informs on the correlation to the most proximal phenotype (45–48), but not directly on the accuracy of the calling on differential chromatin states *per se*. However, we observed that ROTS consistently appeared among the methods reporting highest correlations across the datasets and especially in ATAC-seq data the differential peaks detected by ROTS had stronger correlation with differential transcription compared to the other methods (Figure 5). ROTS also showed higher correlations across all datasets compared to the other two-step methods DiffBind and MAnorm2 with the exception of H3K36me3 dataset, suggesting its potential advantage over DiffBind and MAnorm2 to detect functionally relevant changes. Of the one-step methods THOR displayed stronger correlations than diffReps and PePr. The low correlations shown especially by diffReps were consistent with the observation that the method detected too broad chromatin regions, likely making the fold-change values imprecise. Overall, our analysis suggests that the more accurate detection of peak borders by the two-step methods (with MACS2) was linked to a stronger correlation of the detected differential peaks with differential gene expression levels.

In summary, our study provides evidence that ROTS is a useful addition to the available ChIP-seq differential chromatin modification analysis methods and shows its potential in improving the analysis of differential chromatin states in ATAC-seq data over the currently widely used methods. Moreover, to our knowledge this study is the first comparison that simultaneously tested the differential peak detection methods using both ChIP-seq and ATAC-seq data, serving as a useful reference for the research community increasingly inclined to use ATAC-seq for its ease of use and applicability. The results described here are also of relevance in the analysis of single cell ATAC-seq (scATAC-seq) data where cell type or condition specific clusters may be pooled and analyzed in a similar manner as the bulk ATAC-seq data.

## DATA AVAILABILITY

ROTS (1.16.0) is a Bioconductor R package for differential testing in omics data (https://bioconductor.org/packages/release/bioc/html/ROTS.html).

THOR is part of the regulatory genomic toolbox (0.11.4) software for differential peak calling (https://www.regulatory-genomics.org/rgt/download-installation/)

diffReps (1.55.6) is a software for differential peak calling (https://github.com/shenlab-sinai/diffreps)

PePr (1.1.10) is a software for differential peak calling (https://github.com/shawnzhangyx/PePr)

DiffBind (3.0.15) is a software for differential peak calling (https://bioconductor.org/packages/release/bioc/html/DiffBind.html)

MAnorm2 (1.0.0) is a software for differential peak calling (https://github.com/tushiqi/MAnorm2)

The code used to produce the figures in this study and to perform the differential peak calling with the different methods is available at: https://github/elolab/Faux_et_al_NARGAB2021

The ATAC-seq Yellow-Fever dataset is available on the GEO accession number GSE101609; updated 15 May 2019.

The ATAC-seq IFN dataset is available on the GEO accession number GSE100383; updated 15 May 2019.

The ChIP-seq RA dataset for H3K4me3 and H3K36me3 is available on the GEO accession number GSE112655; updated 19 March 2019.

## Supplementary Material

lqab059_Supplemental_File

## Notes

**Disclaimer:** The funding bodies had no role in the design of the study and data collection, analyses and interpretation of data or in writing of the manuscript.

## References

[1] Baker M. Making sense of chromatin states. Nat. Methods. 2011; 8:717–722. 21878916 10.1038/nmeth.1673

[2] Bannister A.J. , Kouzarides T. Regulation of chromatin by histone modifications. Cell Res. 2011; 21:381–395. 21321607 10.1038/cr.2011.22 PMC3193420

[3] Banerjee S. , Zhu H., Tang M., Feng W., Wu X., Xie H. Identifying transcriptional regulatory modules among different chromatin states in mouse neural stem cells. Front. Genet. 2019; 9:731. 30697231 10.3389/fgene.2018.00731 PMC6341026

[4] Andersson R. , Sandelin A. Determinants of enhancer and promoter activities of regulatory elements. Nat. Rev. Genet. 2020; 21:71–87. 31605096 10.1038/s41576-019-0173-8

[5] Holtzman L. , Gersbach C.A. Editing the epigenome: reshaping the genomic landscape. Annu. Rev. Genomics Hum. Genet. 2018; 19:43–71. 29852072 10.1146/annurev-genom-083117-021632

[6] Kouzarides T. Chromatin modifications and their function. Cell. 2007; 128:693–705. 17320507 10.1016/j.cell.2007.02.005

[7] Boyle A.P. , Davis S., Shulha H.P., Meltzer P., Margulies E.H., Weng Z., Furey T.S., Crawford G.E. High-resolution mapping and characterization of open chromatin across the genome. Cell. 2008; 132:311–322. 18243105 10.1016/j.cell.2007.12.014 PMC2669738

[8] Giresi P.G. , Lieb J.D. Isolation of active regulatory elements from eukaryotic chromatin using FAIRE (Formaldehyde Assisted Isolation of Regulatory Elements). Methods. 2009; 48:233–239. 19303047 10.1016/j.ymeth.2009.03.003 PMC2710428

[9] Buenrostro J.D. , Wu B., Chang H.Y., Greenleaf W.J. ATAC-seq: a method for assaying chromatin accessibility genome-wide. Curr. Protoc. Mol. Biol. 2015; 109:21.29.1–21.29.9. 10.1002/0471142727.mb2129s109 PMC4374986 25559105

[10] Yan H. , Tian S., Slager S.L., Sun Z., Ordog T. Genome-wide epigenetic studies in human disease: a primer on -omic technologies. Am. J. Epidemiol. 2016; 183:96–109. 26721890 10.1093/aje/kwv187 PMC4706679

[11] Zhang Y. , Liu T., Meyer C.A., Eeckhoute J., Johnson D.S., Bernstein B.E., Nussbaum C., Myers R.M., Brown M., Li W. et al. Model-based analysis of ChIP-Seq (MACS). Genome Biol. 2008; 9:R137. 18798982 10.1186/gb-2008-9-9-r137 PMC2592715

[12] Heinz S. , Benner C., Spann N., Bertolino E., Lin Y.C., Laslo P., Cheng J.X., Murre C., Singh H., Glass C.K. Simple combinations of lineage-determining transcription factors prime cis-regulatory elements required for macrophage and B-cell identities. Mol. Cell. 2010; 38:576–589. 20513432 10.1016/j.molcel.2010.05.004 PMC2898526

[13] Divoux A. , Sandor K., Bojcsuk D., Talukder A., Li X., Balint B.L., Osborne T.F., Smith S.R. Differential open chromatin profile and transcriptomic signature define depot-specific human subcutaneous preadipocytes: primary outcomes. Clin. Epigenet. 2018; 10:148. 10.1186/s13148-018-0582-0 PMC6258289 30477572

[14] Yan F. , Powell D.R., Curtis D.J., Wong N.C. From reads to insight: a hitchhiker's guide to ATAC-seq data analysis. Genome Biol. 2020; 21:22. 32014034 10.1186/s13059-020-1929-3 PMC6996192

[15] Steinhauser S. , Kurzawa N., Eils R., Herrmann C. A comprehensive comparison of tools for differential ChIP-seq analysis. Brief. Bioinform. 2016; 17:953–966. 26764273 10.1093/bib/bbv110 PMC5142015

[16] Tu S. , Shao Z. An introduction to computational tools for differential binding analysis with ChIP-seq data. Quant. Biol. 2017; 5:226–235.

[17] Allhoff M. , Seré K., Pires J.F., Zenke M., Costa I.G. Differential peak calling of ChIP-seq signals with replicates with THOR. Nucleic Acids Res. 2016; 44:e153. 27484474 10.1093/nar/gkw680 PMC5175345

[18] Zhang Y. , Lin Y.-H., Johnson T.D., Rozek L.S., Sartor M.A. PePr: a peak-calling prioritization pipeline to identify consistent or differential peaks from replicated ChIP-Seq data. Bioinformatics. 2014; 30:2568–2575. 24894502 10.1093/bioinformatics/btu372 PMC4155259

[19] Shen L. , Shao N.-Y., Liu X., Maze I., Feng J., Nestler E.J. diffReps: detecting differential chromatin modification sites from ChIP-seq data with biological replicates. PLoS One. 2013; 8:e65598. 23762400 10.1371/journal.pone.0065598 PMC3677880

[20] Ross-Innes C.S. , Stark R., Teschendorff A.E., Holmes K.A., Ali H.R., Dunning M.J., Brown G.D., Gojis O., Ellis I.O., Green A.R. et al. Differential oestrogen receptor binding is associated with clinical outcome in breast cancer. Nature. 2012; 481:389–393. 22217937 10.1038/nature10730 PMC3272464

[21] Xu S. , Grullon S., Ge K., Peng W. Spatial clustering for identification of ChIP-enriched regions (SICER) to map regions of histone methylation patterns in embryonic stem cells. Methods Mol. Biol. 2014; 1150:97–111. 24743992 10.1007/978-1-4939-0512-6_5 PMC4152844

[22] Yang Y. , Fear J., Hu J., Haecker I., Zhou L., Renne R., Bloom D., McIntyre L.M. Leveraging biological replicates to improve analysis in ChIP-seq experiments. Comput. Struct. Biotechnol. J. 2014; 9:e201401002. 24688750 10.5936/csbj.201401002 PMC3962196

[23] Allhoff M. , Seré K., Chauvistré H., Lin Q., Zenke M., Costa I.G. Detecting differential peaks in ChIP-seq signals with ODIN. Bioinformatics. 2014; 30:3467–3475. 25371479 10.1093/bioinformatics/btu722

[24] Shao Z. , Zhang Y., Yuan G.-C., Orkin S.H., Waxman D.J. MAnorm: a robust model for quantitative comparison of ChIP-Seq datasets. Genome Biol. 2012; 13:R16. 22424423 10.1186/gb-2012-13-3-r16 PMC3439967

[25] Elo L.L. , Filen S., Lahesmaa R., Aittokallio T. Reproducibility-optimized test statistic for ranking genes in microarray studies. IEEE/ACM Trans. Comput. Biol. Bioinform. 2008; 5:423–431. 18670045 10.1109/tcbb.2007.1078

[26] Seyednasrollah F. , Laiho A., Elo L.L. Comparison of software packages for detecting differential expression in RNA-seq studies. Brief. Bioinform. 2015; 16:59–70. 24300110 10.1093/bib/bbt086 PMC4293378

[27] Suni V. , Seyednasrollah F., Ghimire B., Junttila S., Laiho A., Elo L.L. Reproducibility optimized detection of differential DNA methylation. Epigenomics. 2020; 12:747–755. 32496849 10.2217/epi-2019-0289

[28] Pursiheimo A. , Vehmas A.P., Afzal S., Suomi T., Chand T., Strauss L., Poutanen M., Rokka A., Corthals G.L., Elo L.L. Optimization of statistical methods impact on quantitative proteomics data. J. Proteome Res. 2015; 14:4118–4126. 26321463 10.1021/acs.jproteome.5b00183

[29] Tu S. , Li M., Chen H., Tan F., Xu J., Waxman D.J., Zhang Y., Shao Z. MAnorm2 for quantitatively comparing groups of ChIP-seq samples. Genome Res. 2021; 31:131–145. 33208455 10.1101/gr.262675.120 PMC7849384

[30] Thomas R. , Thomas S., Holloway A.K., Pollard K.S. Features that define the best ChIP-seq peak calling algorithms. Brief. Bioinform. 2017; 18:441–450. 27169896 10.1093/bib/bbw035 PMC5429005

[31] Lun A.T.L. , Smyth G.K *De novo* detection of differentially bound regions for ChIP-seq data using peaks and windows: controlling error rates correctly. Nucleic Acids Res. 2014; 42:e95. 24852250 10.1093/nar/gku351 PMC4066778

[32] Anders S. , Huber W. Differential expression analysis for sequence count data. Genome Biol. 2010; 11:R106. 20979621 10.1186/gb-2010-11-10-r106 PMC3218662

[33] Love M.I. , Huber W., Anders S. Moderated estimation of fold change and dispersion for RNA-seq data with DESeq2. Genome Biol. 2014; 15:550. 25516281 10.1186/s13059-014-0550-8 PMC4302049

[34] Soneson C. , Delorenzi M. A comparison of methods for differential expression analysis of RNA-seq data. BMC Bioinformatics. 2013; 14:91. 23497356 10.1186/1471-2105-14-91 PMC3608160

[35] Robinson M.D. , Oshlack A. A scaling normalization method for differential expression analysis of RNA-seq data. Genome Biol. 2010; 11:R25. 20196867 10.1186/gb-2010-11-3-r25 PMC2864565

[36] Leinonen R. , Sugawara H., Shumway M. International Nucleotide Sequence Database Collaboration The sequence read archive. Nucleic Acids Res. 2011; 39:D19–D21. 21062823 10.1093/nar/gkq1019 PMC3013647

[37] Church D.M. , Schneider V.A., Graves T., Auger K., Cunningham F., Bouk N., Chen H.-C., Agarwala R., McLaren W.M., Ritchie G.R.S. et al. Modernizing reference genome assemblies. PLoS Biol. 2011; 9:e1001091. 21750661 10.1371/journal.pbio.1001091 PMC3130012

[38] Langmead B. , Wilks C., Antonescu V., Charles R. Scaling read aligners to hundreds of threads on general-purpose processors. Bioinformatics. 2019; 35:421–432. 30020410 10.1093/bioinformatics/bty648 PMC6361242

[39] Amemiya H.M. , Kundaje A., Boyle A.P. The ENCODE blacklist: identification of problematic regions of the genome. Sci. Rep. 2019; 9:9354. 31249361 10.1038/s41598-019-45839-z PMC6597582

[40] Li H. , Handsaker B., Wysoker A., Fennell T., Ruan J., Homer N., Marth G., Abecasis G., Durbin R. The Sequence Alignment/Map format and SAMtools. Bioinformatics. 2009; 25:2078–2079. 19505943 10.1093/bioinformatics/btp352 PMC2723002

[41] Wu D.-Y. , Bittencourt D., Stallcup M.R., Siegmund K.D. Identifying differential transcription factor binding in ChIP-seq. Front. Genet. 2015; 6:169. 25972895 10.3389/fgene.2015.00169 PMC4413818

[42] Lawrence M. , Huber W., Pagès H., Aboyoun P., Carlson M., Gentleman R., Morgan M.T., Carey V.J. Software for computing and annotating genomic ranges. PLoS Comput. Biol. 2013; 9:e1003118. 23950696 10.1371/journal.pcbi.1003118 PMC3738458

[43] Johnson W.E. , Li C., Rabinovic A. Adjusting batch effects in microarray expression data using empirical Bayes methods. Biostatistics. 2007; 8:118–127. 16632515 10.1093/biostatistics/kxj037

[44] Suomi T. , Seyednasrollah F., Jaakkola M.K., Faux T., Elo L.L. ROTS: an R package for reproducibility optimized statistical testing. PLoS Comput. Biol. 2017; 13:e1005562. 28542205 10.1371/journal.pcbi.1005562 PMC5470739

[45] Gates L.A. , Foulds C.E., O’Malley B.W. Histone marks in the ‘driver's seat’: functional roles in steering the transcription cycle. Trends Biochem. Sci. 2017; 42:977–989. 29122461 10.1016/j.tibs.2017.10.004 PMC5701853

[46] Karlić R. , Chung H.-R., Lasserre J., Vlahovicek K., Vingron M. Histone modification levels are predictive for gene expression. Proc. Natl Acad. Sci. U.S.A. 2010; 107:2926–2931. 20133639 10.1073/pnas.0909344107 PMC2814872

[47] Starks R.R. , Biswas A., Jain A., Tuteja G. Combined analysis of dissimilar promoter accessibility and gene expression profiles identifies tissue-specific genes and actively repressed networks. Epigenet. Chromatin. 2019; 12:16. 10.1186/s13072-019-0260-2 PMC6385419 30795793

[48] Toenhake C.G. , Fraschka S.A.-K., Vijayabaskar M.S., Westhead D.R., van Heeringen S.J., Bártfai R. Chromatin accessibility-based characterization of the gene regulatory network underlying plasmodium falciparum blood-stage development. Cell Host Microbe. 2018; 23:557–569. 29649445 10.1016/j.chom.2018.03.007 PMC5899830

[49] Jangid R.K. , Kelkar A., Muley V.Y., Galande S. Bidirectional promoters exhibit characteristic chromatin modification signature associated with transcription elongation in both sense and antisense directions. BMC Genomics. 2018; 19:313. 29716520 10.1186/s12864-018-4697-7 PMC5930751

[50] Cui P. , Liu W., Zhao Y., Lin Q., Ding F., Xin C., Geng J., Song S., Sun F., Hu S. et al. The association between H3K4me3 and antisense transcription. Genomics Proteomics Bioinform. 2012; 10:74–81. 10.1016/j.gpb.2012.05.001 PMC5054153 22768981

[51] Castelnuovo M. , Zaugg J.B., Guffanti E., Maffioletti A., Camblong J., Xu Z., Clauder-Münster S., Steinmetz L.M., Luscombe N.M., Stutz F. Role of histone modifications and early termination in pervasive transcription and antisense-mediated gene silencing in yeast. Nucleic Acids Res. 2014; 42:4348–4362. 24497191 10.1093/nar/gku100 PMC3985671

[52] Akondy R.S. , Fitch M., Edupuganti S., Yang S., Kissick H.T., Li K.W., Youngblood B.A., Abdelsamed H.A., McGuire D.J., Cohen K.W. et al. Origin and differentiation of human memory CD8 T cells after vaccination. Nature. 2017; 552:362–367. 29236685 10.1038/nature24633 PMC6037316

[53] Park S.H. , Kang K., Giannopoulou E., Qiao Y., Kang K., Kim G., Park-Min K.-H., Ivashkiv L.B. Type I interferons and the cytokine TNF cooperatively reprogram the macrophage epigenome to promote inflammatory activation. Nat. Immunol. 2017; 18:1104–1116. 28825701 10.1038/ni.3818 PMC5605457

[54] Ai R. , Laragione T., Hammaker D., Krishna V., Pocalyko D., Whitaker J.W., Bai Y., Nagpal S., Bachman K.E., Ainsworth R.I. et al. Comprehensive epigenetic landscape of rheumatoid arthritis fibroblast-like synoviocytes. Nat Commun. 2018; 9:1921. 29765031 10.1038/s41467-018-04310-9 PMC5953939

