# Supplementary material for: Differential ATAC-seq and ChIP-seq peak detection using ROTS
Source: NAR Genom Bioinform. 2021 Jul 2;3(3):lqab059. doi: 10.1093/nargab/lqab059 (PMC8253552; doi:10.1093/nargab/lqab059)
Supplement: lqab059_Supplemental_File [file lqab059_supplemental_file.pdf]

Supplementary file: Figure S1 – Figure S5

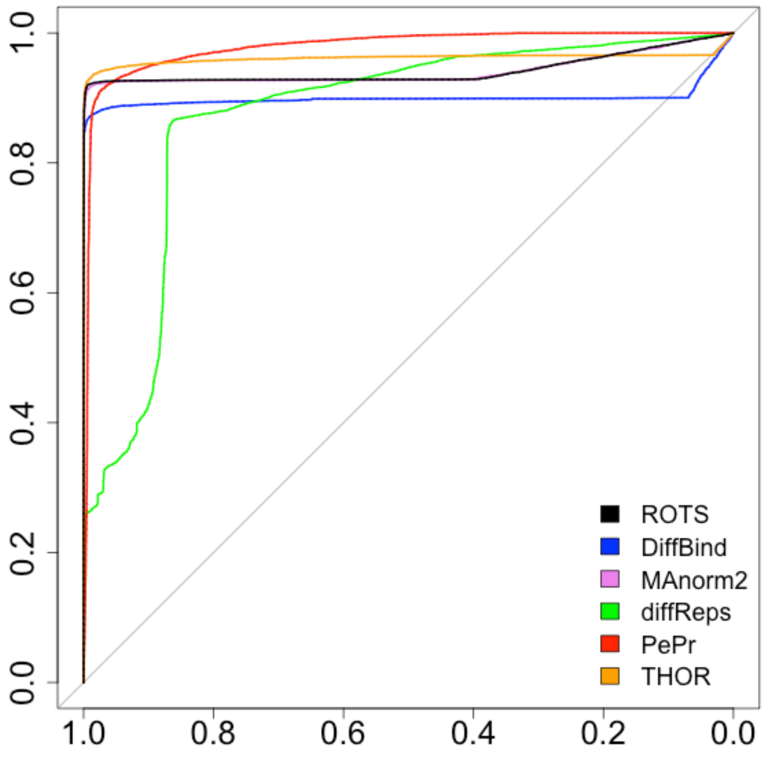

Figure S1: Sensitivity and specificity of the methods displayed by receiver operating characteristic (ROC) curves.

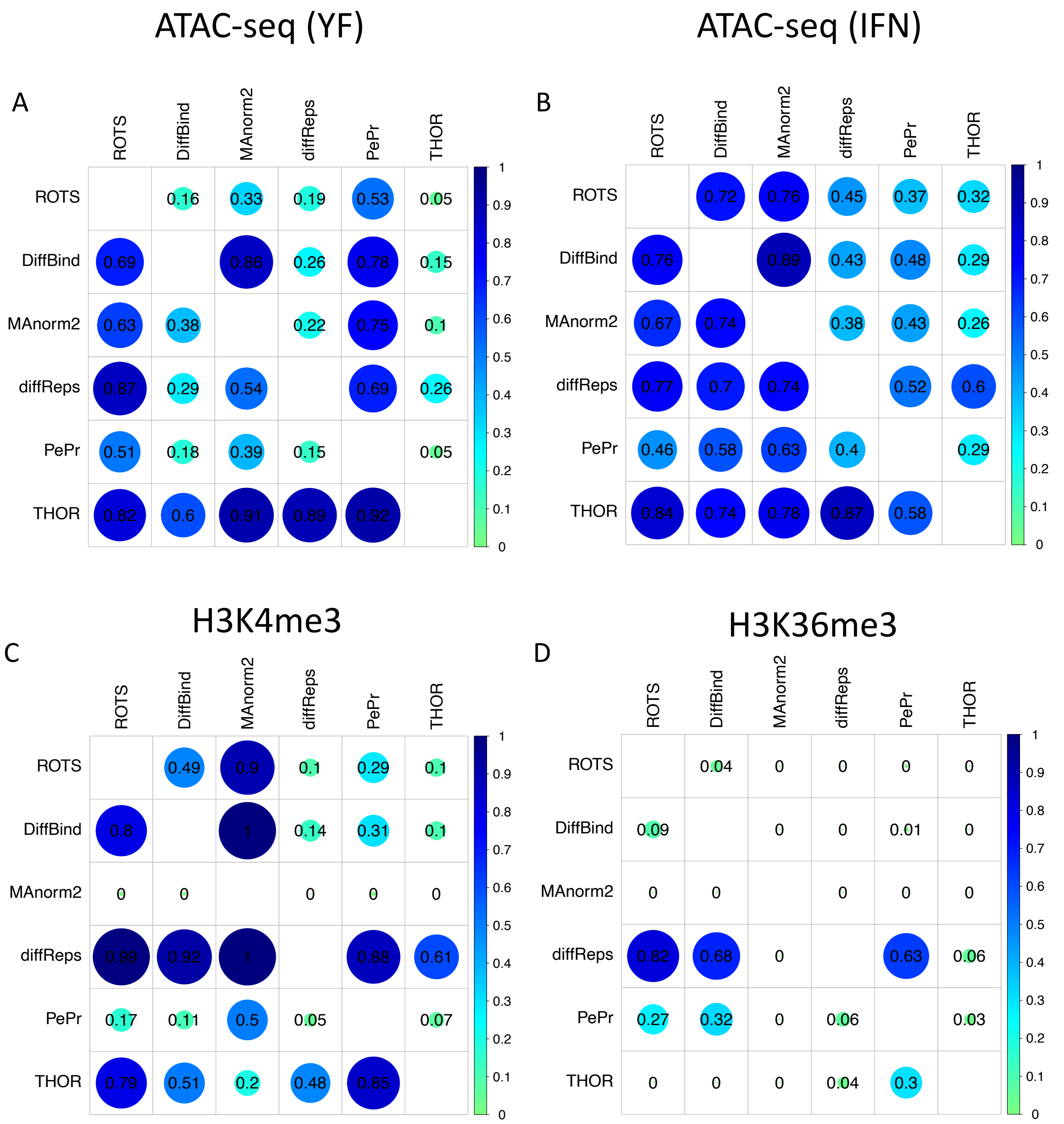

Figure S2. Overlap of significant detections (FDR<0.05) across the methods and datasets. Proportion of detected peaks overlapping between each pair of methods in A) Yellow Fever ATAC-seq, B) Interferon response ATAC-seq, C) Rheumatoid arthritis H3K4me3 ChIP-seq, and D) Rheumatoid arthritis H3K36me3 ChIP-seq dataset.

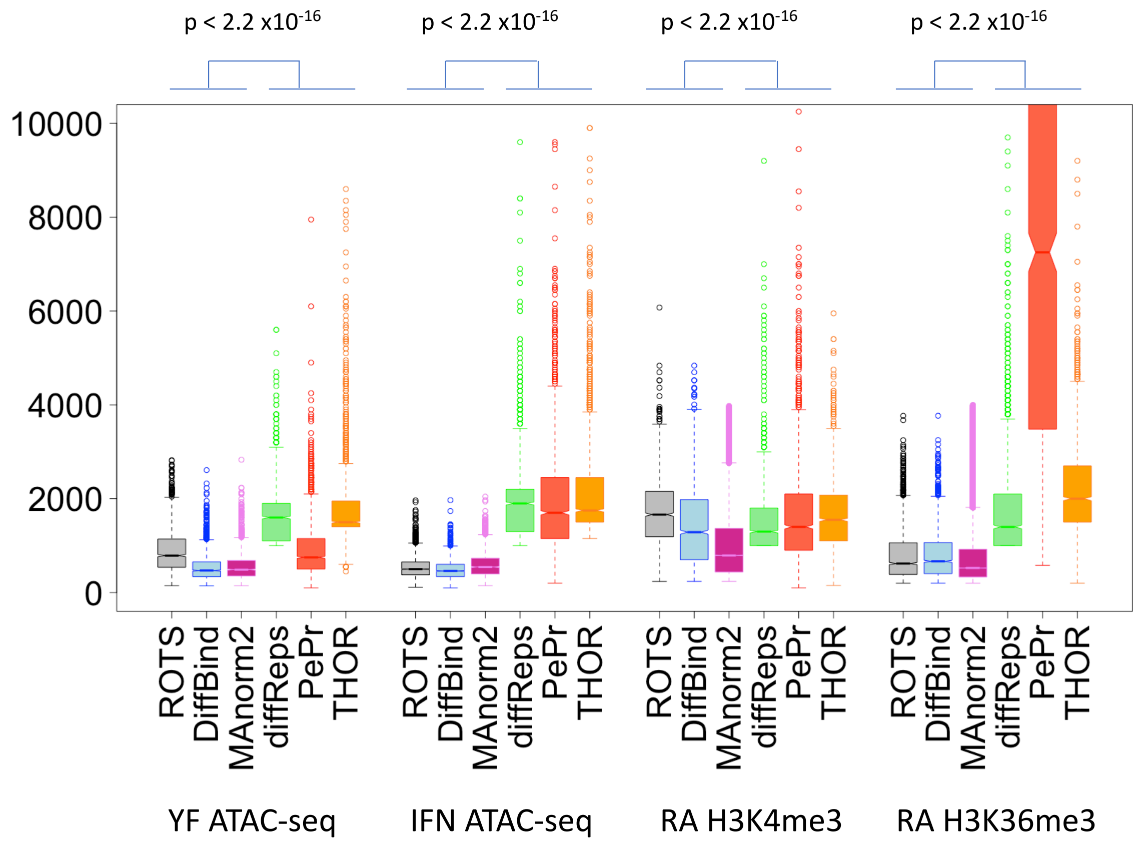

Figure S3. Boxplot of the peak width distribution for each biological dataset across the methods.

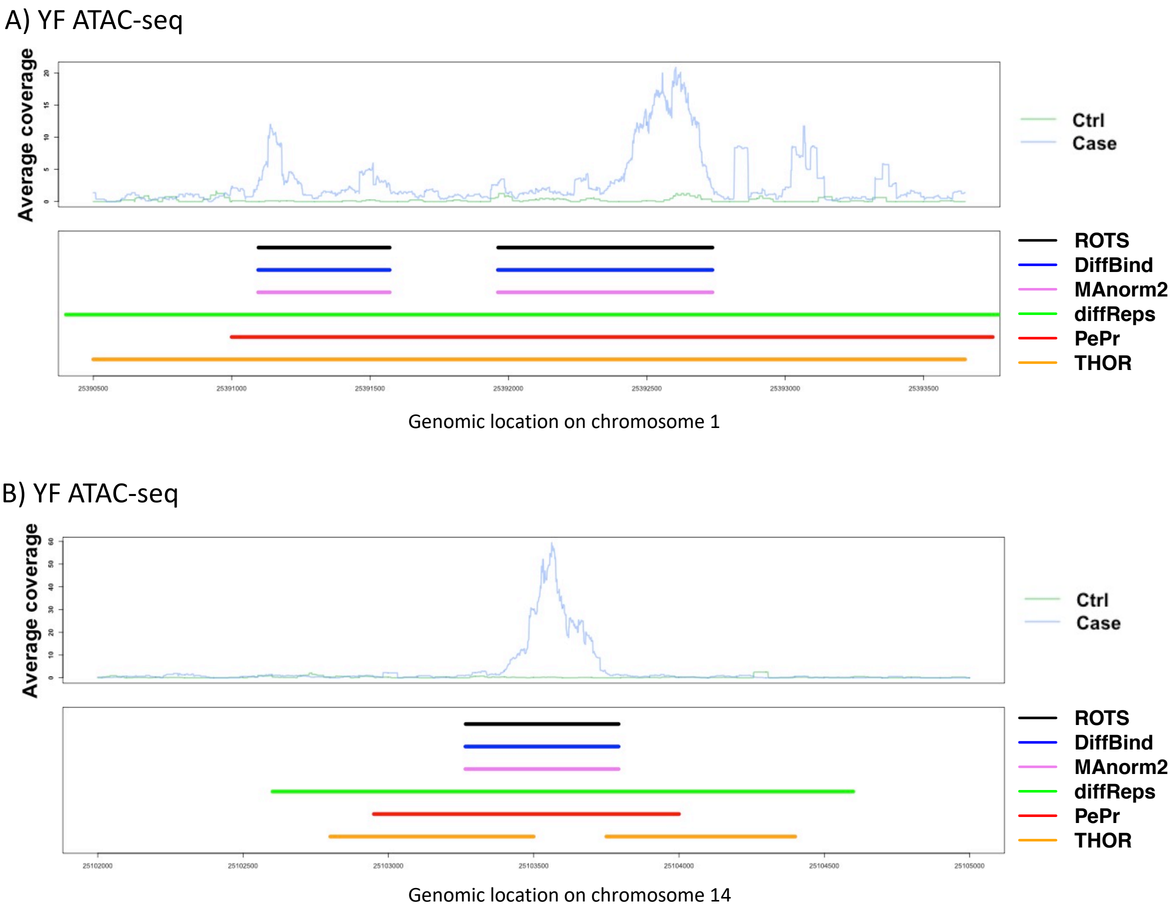

Figure S4. Selected representative examples of detected differential peaks in the genomic context from the Yellow Fever ATAC-seq dataset. Top panels display the average read count over each biological condition and bottom panels mark the detected differential peak region with each method.

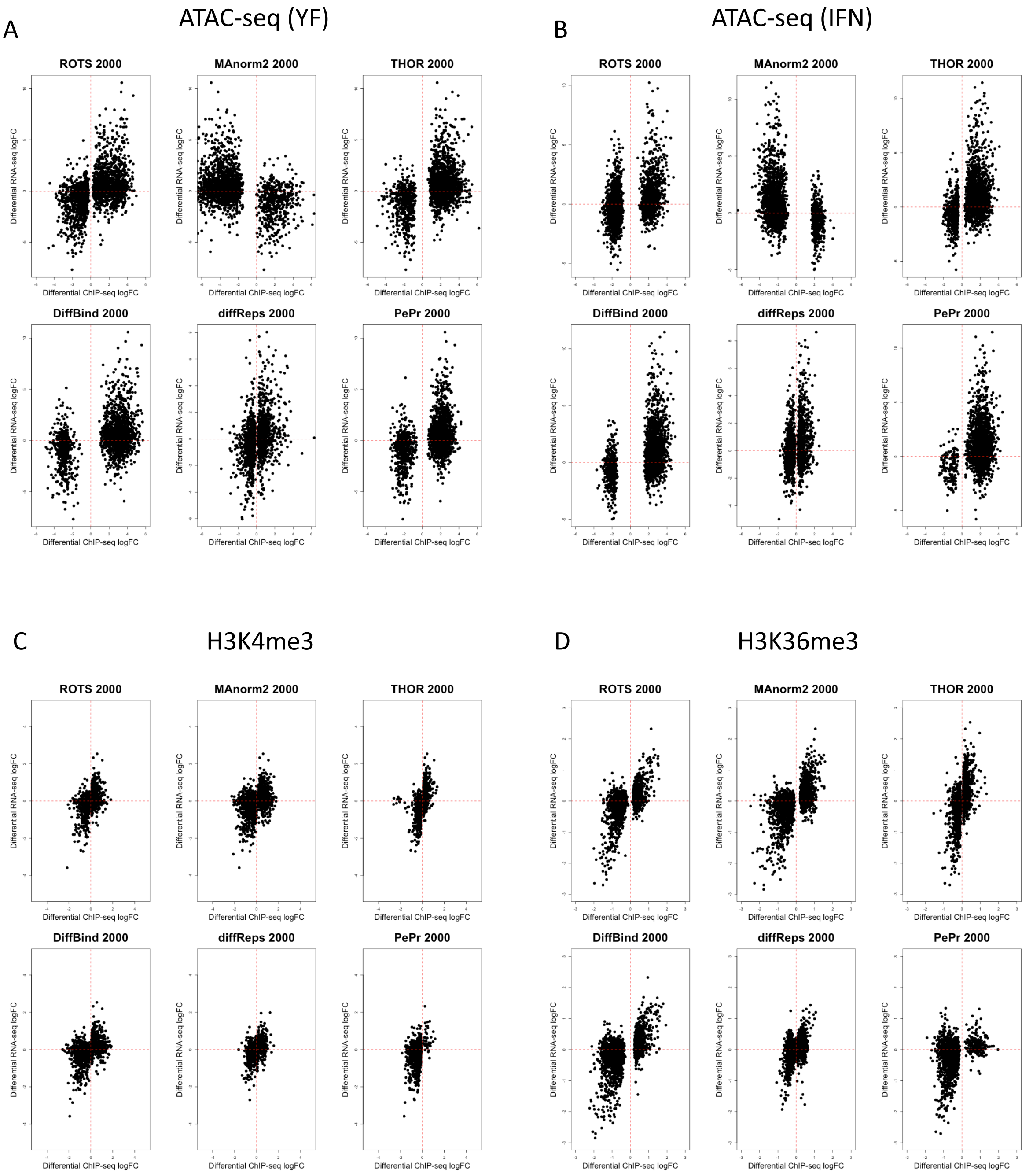

Figure S5. Pearson correlation analysis of differential peak fold-change and gene expression fold-change. Scatterplot presentations of the top 2000 significant differential peaks. The global Pearson correlation is calculated for A) Yellow Fever ATAC-seq, B) Interferon response ATAC-seq, C) Rheumatoid arthritis H3K4me3 ChIP-seq, and D) Rheumatoid arthritis H3K36me3 ChIP-seq dataset.
